# Association of CDSS score and 60-day mortality in Chinese patients with non-APL acute myeloid leukemia: a retrospective cohort study

**DOI:** 10.1007/s11239-023-02850-6

**Published:** 2023-06-23

**Authors:** Huaqing Zhong, Xianchun Chen, Dejun Xiao, Shi Luo, Yanhong Ji, Zuomiao Xiao

**Affiliations:** 1 Central Hospital of Fenggang Town, Ganzhou Economic and Technological Development Zone, Ganzhou, Jiangxi 341412 People’s Republic of China; 2 Department of Clinical Laboratory, The Affiliated Ganzhou Hospital of Nanchang University, Ganzhou, Jiangxi 341000 People’s Republic of China; 3 Department of Immunology and Microbiology, School of Medicine, Xi’an Jiaotong University, Xi’an, Shaanxi 710049 People’s Republic of China

**Keywords:** Acute myeloid leukemia (AML), Non-acute promyelocytic leukemia (non-APL), Disseminated intravascular coagulation (DIC), Chinese DIC scoring system (CDSS), 60-day mortality

## Abstract

Acute myeloid leukemia (AML) is a heterogeneous hematological malignancy, and is one of the triggers of DIC, the latter is an essential factor in the early death of patients with AML. However, the timely identification of DIC remains a challenge. The Chinese DIC Scoring System (CDSS) is a common consensus widely used in China; but, there are few reports on its application in patients with AML. We undertake this retrospective cohort study to investigate the association between CDSS score and 60-day mortality. CDSS scores were evaluated after admission. The outcome was all-cause 60-day mortality. Multivariate Cox regression analyses were performed to calculate the adjusted hazard ratio (HR) and the corresponding 95% confidence interval (CI). Survival curves were plotted by Kaplan–Meier and log-rank analyses. Subgroup analyses were stratified by relevant effect covariates. A total of 570 consecutive patients with primary AML were included. We found an association between a 39% increase in 60-day mortality and a 1 point increase in CDSS score (HR = 1.39, 95% CI 1.25–1.54), which was associated with a 189% increase in 60-day mortality in CDSS scores ≥ 6 compared with that in the CDSS scores < 6 (HR = 2.89, 95% CI 1.91–4.38). After adjusting for all potential con-founders, a 27% and a 198% increase were observed (HR = 1.27, 95% CI 1.01–1.61; HR = 2.98, 95% CI 1.24–7.19), respectively. There is association between 60-day mortality and CDSS score in patients with AML. These findings may help hematologists in making informed treatment decisions.

**Supplementary Information:**

The online version contains supplementary material available at 10.1007/s11239-023-02850-6.

## Highlights

- The CDSS score may be closely associated with 60-day mortality in patients with primary non-APL AML.
- These results, which are of great clinical significance in patients with CDSS scores ≥ 6, suggest that hematologists should quickly start anti-DIC and anti-leukemia treatments.

## Introduction

Acute myeloid leukemia (AML), one of the most common type of hematological malignancies in adults, is a heterogeneous clonal myeloid neoplasm characterized by maturation arrest of hematopoietic progenitor cells, leading to uncontrolled blast proliferation [1–3]. Abnormal differentiation of myeloid cells results in high levels of immature malignant cells and low levels of healthy hematopoietic elements. Cytopenia causes clinical manifestations, with symptoms of anemia (e.g., dyspnea and fatigue), thrombocytopenia (hemorrhage), and neutropenia (infections), which are usually present at the time of diagnosis and accompanied by treatment [4].

The early mortality was 21.0–37.5% reported in previous studies [5–7], although hematologists have taken much effort into reducing 60-day mortality, commonly known as early death, defined as death from any cause within 60 days of hospitalization with AML [7, 8],it remains a vital clinical problem to be solved [9]. Disseminated intravascular coagulation (DIC) is a systemic activation of the coagulation system that results in microvascular thrombosis and, simultaneously, potentially life-threatening hemorrhage attributed to the consumption of platelets and coagulation factors. Underlying conditions, including hematological malignancies and infection, are responsible for the initiation and propagation of DIC[10, 11]. Patients with AML are prone to co-infection, both AML and infection are trigger factors of DIC. DIC, considered a clinical laboratory diagnosis by thrombosis and hemostasis specialists, is complicated [12]. Patients with DIC tend to stay in critical condition and have high mortality rates. It is still a challenging task requiring professional experience to diagnose DIC in patients with AML accurately [13]. In China, the latest edition of the consensus of Chinese experts on DIC diagnosis published in 2017 has been widely accepted by physicians and investigators caring for DIC patients [14, 15]. However, the association between the Chinese Disseminated Intravascular Coagulation Scoring System (CDSS) score and 60-day mortality is uncertain, and only limited data are available on the application of the CDSS for diagnosing DIC in patients with non-APL AML [16].This study aimed to investigate the association between CDSS scores and 60-day mortality in primary AML.

## Methods

This is a cohort study based on retrospectively collected consecutive data from patients of all ages, verified primary non-APL AML at the Affiliated Ganzhou Hospital of Nanchang University (Jiangxi Province, China) from January 2013 to July 2022. All individuals in this study underwent bone marrow (BM) aspiration and had confirmed AML diagnosis based on more than two methods of morphological, immunophenotype, cytogenetic, and molecular analysis (MICM), according to the World Health Organization (WHO) classification system (version 2016) [17] The patients were classified with genomic risk category, which were quired by karyotype analysis (G-banding), fluorescence in situ hybridization (FISH) and next generation sequencing (NGS) [1, 18]. This study followed the principles of the Declaration of Helsinki and was approved (Ethics number: 202005) by the Ethics Review Board of the Affiliated Ganzhou Hospital of Nanchang University [19].

Patients diagnosed with acute promyelocytic leukemia (APL) were excluded, due to different management and treatment for APL and non-APL AML [20, 21]. Patients with a history of other hematological malignancies, such as chronic myeloid leukemia (CML), myelodysplastic syndrome (MDS), and myelodysplastic syndrome/ myeloproliferative neoplasms (MDS/MPN), were excluded due to secondary AML, read our previous report in brief [19]. Patients with the following criteria were also excluded: thrombotic thrombocytopenic purpura (TTP) based on clinical history, examination and routine laboratory parameters, having anti-phospholipid syndrome (APS) based on at least one of the clinical and one of the laboratory criteria, liver cirrhosis classified as Child–Pugh grade C (score 10 or higher). These primary diseases might lead to abnormal platelet (PLT), D-dimer (DD), or fibrinogen (Fg) and could affect CDSS scores. Mixed phenotype acute leukemia (MPAL) was also excluded due to the strict sense of patients with non-AML [17].

**Fig. 1:**
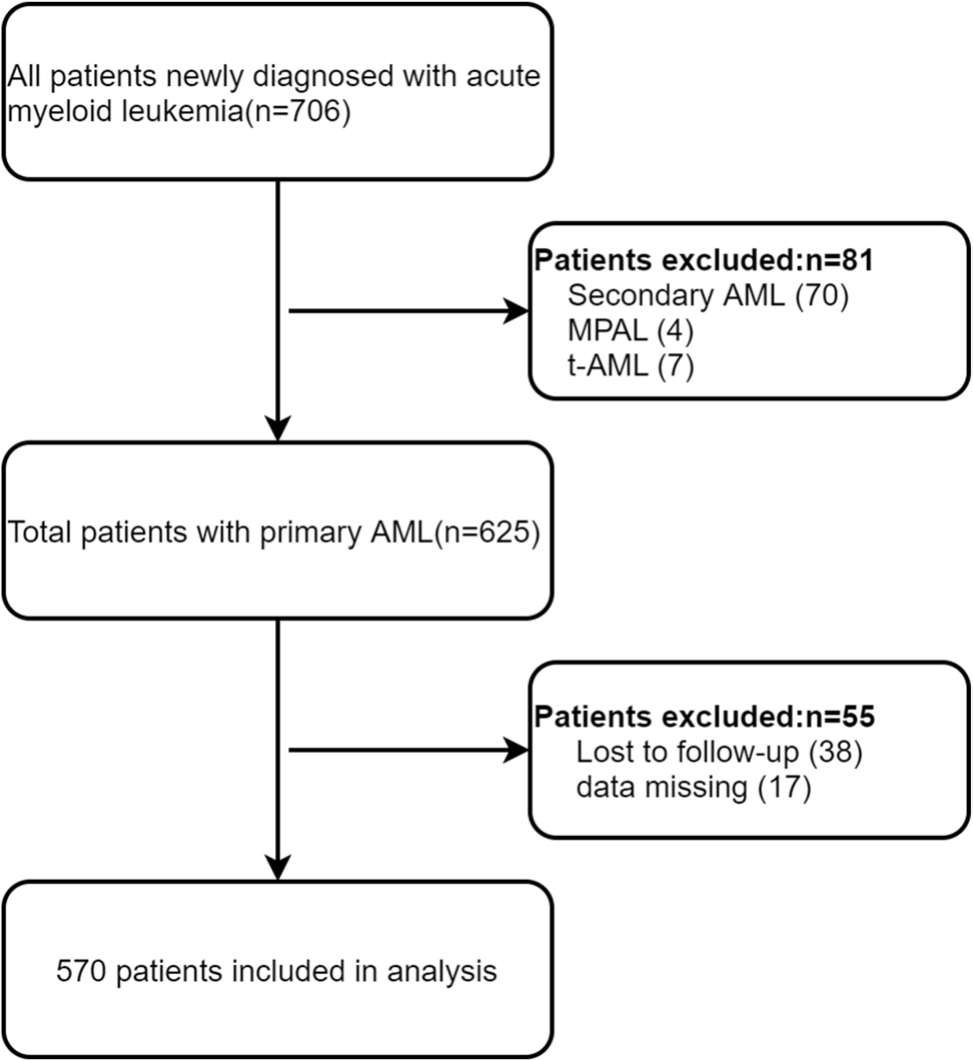
Flowchart of the patient selection process. Abbreviations: AML, acute myeloid leukemia; MPAL, mixed phenotype acute leukemia; MDS, myelodysplastic syndrome; t-AML, therapy-related AML

In addition, patients did not undergo complete CDSS test items or were lost to follow-up were excluded; all remaining patients were included in the study with or without chemotherapy (Fig. 1). None of the patients underwent bone marrow transplantation 60 days after admission. The final cohort included 570 patients with AML classified into three subtypes: AML-M_2_, AML-M_5,_ and the other subgroup. The other subgroup included 127 patients classified as follows: AML-M_0_ (n = 5), AML-M_1_ (n = 40), AML-M_4_ (n = 68), AML-M_6_ (n = 4), and AML-M_7_ (n = 10). (Fig. 1).

### CDSS score

The latest CDSS was established in 2017 by a consensus of Chinese experts on the diagnosis and treatment of DIC [14]. According to this consensus, DIC is diagnosed when a patient with hematologic malignancies has abnormal laboratory findings [14].This consensus assigns quantitative weights to individual laboratory and clinical parameters for DIC diagnosis. The CDSS criteria includes following five rules. (1) A primary disease that causes DIC, hematologic malignancy e.g.is present, 2 points. (2) Prolongation of PT and APTT: PT < 3S and APTT < 10 S,0 point; PT ≥ 3S and APTT ≥ 10 S,1 point; PT > 6S, 2 points. (3)D-dimer level: < 5 μg/mL, 0 point; ≥ 5 μg/mL, 2 points; ≥ 9 μg/mL, 3 points. (4) Fibrinogen level: ≥ 1.0 g/L,0 point; < 1.0 μg/mL, 1point. (5) Platelet counts: > 50 × 10^9^/L,0 point; Platelet counts < 50 × 10^9^/L,1 point; Diagnosis of DIC is CDSS score ≥ 6 points for AML Patients (Table S1). All, except PLT count, were measured using the blood coagulation analyzer Sysmex CS-5100 System TM (Siemens Healthcare Diagnostics, Erlangen, Germany) with the corresponding diagnostic reagents kit (Siemens Healthcare Diagnostics Products, Germany, Marburg, Germany). and the reference interval for PT was 9–13 s, APTT 20–40 s, Fib 2–4 g/L, and DD 0–0.55 mg/L, in accordance with the national standard. Commercially available control materials for internal quality control (IQC) (Siemens Healthcare Diagnostics Products, Marburg, Germany) were used to test blood samples daily. PLT counts were obtained from routine blood samples, measured using a Sysmex XE-2100 or XN-1000 s. All five items, including the CDSS score, were included in external quality assessment (EQA) activities organized by the National Center for Clinical Laboratories (NCCL) every year, and criteria for feedback reports were fulfilled during this study. All data (except PLT) from this study, including CDSS and other items/parameters, were the first test results of patients after admission for AML. If the CDSS score of the patients was ≥ 6, they were diagnosed with overt DIC, and if the CDSS score was 5 points with PLT ≥ 50 × 10^9^/L for the first time, another two PLT count were required for monitoring; if the PLT decreased by 50% within 48 h, the CDSS score would be calculated repeatedly.

### Outcomes

The primary endpoint and outcome of interest were death within 60 days of admission. The data collectors for clinical information at the first diagnosis were blinded to the survival data.

### Data collection

Data, including survival status, were collected from the electronic medical record system or follow-up telephone calls. Baseline examinations included FAB subtype, genomic risk category, bleeding and thrombosis, pulmonary infection, blood and bone marrow (BM) parameters. The biomarkers included white blood cell (WBC) count, PLT count, prothrombin time (PT), activated partial thromboplastin time (APTT), DD, Fg, creatine kinase isoenzyme-MB (CK-MB), myoglobin (Myo), antithrombin (AT), albumin (Alb), creatinine (Crea), glucose (Glu), serum ferritin (SF), and BM blast. All laboratory data were focused on measurements performed within the first 48 h of admission to reduce the probability that serum biomarker levels were affected by anti-leukemia and transfusion therapy. These parameters comprise routine testing, which physicians commonly use to evaluate a patient’s physical condition. The chemotherapy was completed within 60 days.

### Statistical analysis

This study aimed to observe the impact of CDSS score on 60-day mortality in patients with AML. The patients were divided into two groups based on their CDSS scores. A descriptive analysis was applied to all patients. Continuous data were expressed as mean and standard deviation (SD) or median and interquartile range (quartile 1–quartile 3 [interquartile range (IQR)], as appropriate. Categorical variables were expressed as proportions (%). Variables were compared using the chi-square test (categorical variables), one-way ANOVA (normal distribution), and Kruskal–Wallis (skewed distribution) tests [19].

Multivariate Cox regression analysis was used to assess the independent association between CDSS score and 60-day mortality. In the analysis, confounding factor is an important issue, we performed some different statistical models to verify the results’ stability. In the final model, we adjusted the factors basing the following two rules. (1) For univariate analysis, we adjusted for variables, of which the p values were less than 0.1(Table S2). (2) For multivariate analysis, variables were chosen on the basis of previous findings and clinical constraints. Dummy variables were used to indicate the missing covariance values, if the missing data variables were greater than 10%. Considering the strong heterogeneity of WBC in leukemia, WBC were used as a dichotomous variable to adjust in multivariate Cox regression analysis.

Survival curves were plotted by Kaplan–Meier and log-rank analyses. Subgroup analyses were stratified by relevant effect covariates. Analyses were stratified according to the results of the univariate analysis (P-value < 0.1) and basis of previous findings and clinical constraints, including sex, age, WBC, Glu, ALB, chemotherapy and bleeding, to examine the effect of these factors on the above associations. The likelihood ratio test was used to assess effect modification according to prespecified subgroups using interaction terms between subgroup indicators and CDSS scores. Interactions across subgroups were tested using the likelihood ratio test. All analyses were performed using R 3.3.2 (http://www.R-project.org, The R Foundation) and Free Statistics (version 1.5). Differences with a two-sided P < 0.05 were considered statistically significant [22].

## Results

### Baseline characteristics of the study participants by categories of CDSS score

Of the 706 newly diagnosed patients with AML during the study period, 570 ones were eligible for analysis (Fig. 1). The descriptive characteristics of the eligible study population with a CDSS < 6 and ≥ 6 are reported in Table 1. Patient age was 51.3 (SD 20.4) years (range, 1–94 years), 306 (53.7%) were male, and 137 (24.0%) suffered from 60-day mortality. In addition, 342 (60.1%) patients received combined chemotherapy, and 92 (16.2%) patients single chemotherapy drugs. The combined chemotherapy regimens were a combination of cytarabine and anthracycline “7 + 3” or a combination of cytarabine and the other, 7 patients were treated with the combination of cytarabine and venetoclax. Single chemotherapy meant patients only used decitabine or hydroxyurea [23]. The median CDSS score level was 3.4 ± 1.4 points (range, 0–9 points). 59 (10.4%) cases had CDSS scores ≥ 6 points. The 60-day mortality of patients with CDSS score ≥ 6 was as high as 47.5%, significantly higher than those < 6 at 21.3% (P < 0.001).

**Table 1:** Baseline characteristics of the study participants

Variables	Total	CDSS score < 6	CDSS score ≥ 6	*P* value
	(n = 570)	(n = 511)	(n = 59)	
Sex, n (%)				0.928
Male	306 (53.7)	274 (53.6)	32 (54.2)	
Female	264 (46.3)	237 (46.4)	27 (45.8)	
Age(years)	51.3 ± 20.4	51.1 ± 20.1	52.9 ± 22.8	0.510
FAB subtype, n (%)				0.141
AML-M_2_	251 (44.0)	232 (45.4)	19 (32.2)	
AML-M_5_	192 (33.7)	167 (32.7)	25 (42.4)	
Other	127 (22.3)	112 (21.9)	15 (25.4)	
Genomic risk category, n (%)				0.023
Low	88 (22.3)	85 (23.5)	3 (9.1)	
Medium	171 (43.4)	159 (44)	12 (36.4)	
High	135 (34.3)	117 (32.4)	18 (54.5)	
Bleeding, n (%)				0.721
Without	349 (61.2)	316 (61.8)	33 (55.9)	
Skin or Mucosa	177 (31.1)	155 (30.3)	22 (37.3)	
Internal organs	33 (5.8)	30 (5.9)	3 (5.1)	
Thrombosis	11 (1.9)	10(2.0)	1(1.7)	
Pulmonary infection, n (%)				0.057
No	217 (38.5)	201 (39.9)	16 (27.1)	
Yes	346 (61.5)	303 (60.1)	43 (72.9)	
Chemotherapy, n (%)				0.001
Without	135 (23.7)	114 (22.3)	21 (36.2)	
Combined	342 (60.1)	320 (62.6)	22 (37.9)	
Single	92 (16.2)	77 (15.1)	15 (25.9)	
60-day mortality, n (%)				< 0.001
No	433 (76.0)	402 (78.7)	31 (52.5)	
Yes	137 (24.0)	109 (21.3)	28 (47.5)	
WBC (× 10^9^/L)	19.7 (4.5, 61.9)	16.5 (4.2, 51.0)	79.6 (26.2, 160.2)	< 0.001
PLT (× 10^9^/L)	36.0 (18.0, 72.0)	39.0 (19.0, 79.0)	22.0 (12.0, 34.5)	< 0.001
PT (S)	13.1 (12.2, 14.4)	12.9 (12.1, 14.2)	15.2 (13.8, 16.6)	< 0.001
APTT (S)	29.4 (26.0, 34.6)	29.0 (25.9, 34.0)	33.4 (29.2, 38.8)	< 0.001
Fg (g/L)	3.6 ± 1.5	3.7 ± 1.4	2.3 ± 1.5	< 0.001
DD (mg/L)	1.5 (0.7, 4.8)	1.2 (0.7, 3.1)	12.7 (9.3, 23.0)	< 0.001
AT (%)	87.3 ± 17.5	88.0 ± 17.0	81.6 ± 20.4	0.008
Alb (g/L)	36.8 ± 5.2	36.9 ± 5.2	35.8 ± 5.2	0.126
Crea (umol/L)	69.0 (55.5, 87.8)	68.3 (55.0, 85.0)	73.0 (60.0, 125.0)	0.002
TG (mmol/L)	1.4 (1.0, 1.8)	1.4 (1.0, 1.8)	1.4 (1.1, 1.9)	0.444
HDL (mmol/L)	0.8 (0.6, 1.0)	0.8 (0.6, 1.1)	0.7 (0.5, 0.9)	0.003
Glu (mmol/L)	6.4 ± 2.3	6.4 ± 2.3	6.8 ± 2.3	0.180
CK-MB (U/L)	10.7 (6.9, 17.4)	10.5 (6.7, 16.8)	12.6 (9.5, 29.3)	0.005
SF (ug/L)	662.8 (376.2, 1219.0)	642.2 (360.7, 1158.0)	1089.0 (582.4, 1636.0)	< 0.001
Myo (ng/mL)	20.6 (16.9, 32.4)	20.1 (16.9, 30.6)	28.9 (19.5, 87.8)	0.005
BM blast(%)	58.3 ± 23.1	57.1 ± 22.8	68.3 ± 24.3	< 0.001
CDSS (point)	3.4 ± 1.4	3.0 ± 0.9	6.4 ± 0.7	< 0.001

Data presented are mean ± SD, median (Q1-Q3), or N (%)

*FAB* French, American, British, *WBC* white blood cell, *PLT* platelet, *PT* prothrombin time, *APTT* activated partial thromboplastin time, *Fg* fibrinogen, *DD* D-dime, *AT* antithrombin, *ALB* albumin, *Crea* creatinine, *TG* triglyceride, *HDL* high-density lipoprotein, *Glu* glucose, *CK-MB* creatine kinase isoenzyme MB, *SF* serum ferritin, *Myo* myoglobin, *BM* bone marrow, *CDSS* Chinese DIC scoring system, *DIC* disseminated intravascular coagulation

Additionally, among CDSS laboratory integral items, the PLT lower than 50 × 10^9^/L had the highest abnormal rate with 61.1% (348 cases), followed by the D-dimer (≧5.0 mg/L) 24.2% (138 cases). There were 8 cases (1.4%) with prolongation of APTT (≧10 S) and 51 cases (8.9%) that of PT prolongation (≧3 S). Among 59 patients with overt DIC, the abnormality rate of DD was as high as 96.6% (57 cases, including DD ≥ 5 mg/L in 11 cases and ≥ 9 mg/L in 46 cases), followed by PLT < 50 × 10^9^/L in 93.2% (55 cases), prolongation of PT (≥ 3S), 40.7% (24 cases), Fg(< 1.0 g/L) 18.6%(11 cases), prolongation of APTT (≥ 10S) 4.1% (4 cases).

We evaluated the cohort with ISTH creteria, and found there were 89 (15.6%) patients who had elevated ISTH scores (≥ 5) diagnostic of DIC, with early mortality 42.7%, among those patients there were 58 ones with CDSS score ≥ 6, 28 patients suffered from early death. The morbidity of DIC was 10.4% according to creteria of CDSS, which was lower than that of ISTH one, but the mortality was 47.5%, higher than the former.

### Association between CDSS score and 60-day mortality

**Fig. 2:**
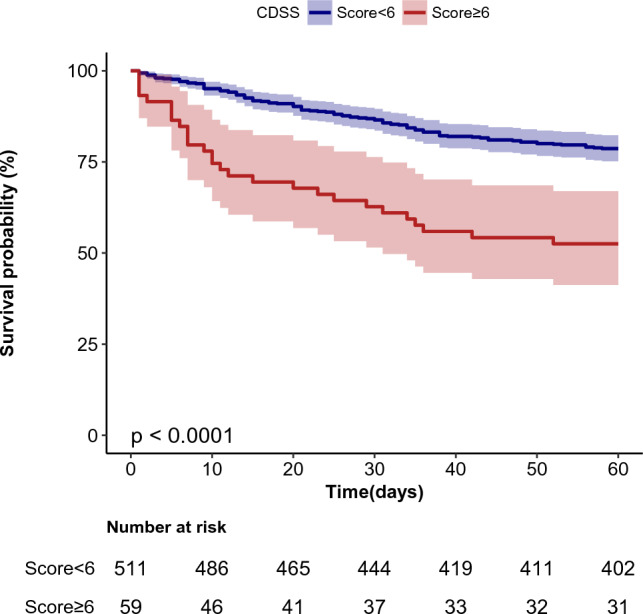
Kaplan–Meier survival curves for day 60 of patients with AML

In the extended multivariate Cox models (Table 2), we observed that the hazard ratios (HRs) of the CDSS score (increase per 1 point) were consistently significant in all three models (HRs range 1.26–1.39). Patients with a CDSS score ≥ 6 (overt DIC) had a 189% higher risk of 60-day mortality than patients with a CDSS score < 6 (non-overt DIC) (HR = 2.89, 95% confidence interval (CI) 1.91–4.38). After adjusted for all covariates, results showed a 198% higher 60-day mortality rate in patients with a CDSS score ≥ 6 (HR = 2.98, 95% CI 1.24–7.19, model III, Table 2) than in those with that < 6. The covariates were confirmed after a univariate analysis of risk factors with a *P*-value ≤ 0.027 (Table S2). Dummy variables were used for serum ferritin due to its missing data was14%. In this observation population, WBC ranged from 0.29 × 10^9^/L to 540.52 × 10^9^/L, with a median of 19.7 × 10^9^/L. We divided WBC into two groups with 20 × 10^9^/L, then used binary WBC as one of the adjusted variables of multivariate Cox regression analysis. We also analyzed the data using the International Society on Thrombosis and Hemostasis (ISTH) criteria and obtained similar results (Table S3). The Kaplan–Meier curve showed that mortality was higher by day 60 in patients with a CDSS score ≥ 6 (log-rank test:* P* < 0.0001, Fig. 2).

**Table 2:** Multivariate Cox regression for CDSS score on 60-day mortality of AML

Variable	Non-adjusted Model		Model I		Model II		Model III	
	HR (95% CI)	*P*-value	HR (95% CI)	*P*-value	HR (95% CI)	*P-*value	HR (95% CI)	*P-*value
CDSS score	1.39 (1.25 ~ 1.54)	< 0.001	1.39 (1.25 ~ 1.54)	< 0.001	1.26 (1.06 ~ 1.49)	0.009	1.27 (1.01 ~ 1.61)	0.045
Binary variable								
CDSS score < 6	Ref		Ref		Ref		Ref	
CDSS score ≥ 6	2.89 (1.91 ~ 4.38)	< 0.001	2.77 (1.82 ~ 4.20)	< 0.001	2.58 (1.36 ~ 4.88)	0.004	2.98 (1.24 ~ 7.19)	0.013

Model I: Adjusts for sex + age; Model II: adjusts for Model I + FAB subtype + bleeding + prognostic stratification + Pulmonary infection + chemotherapy; Model III: adjusts for Model II + BM blast + AT + ALB + Crea + HDL + TG + Glu + CK-MB + Myo + SF + WBC. Dummy variables were used for SF; WBC was entered as a categorical variable

*CDSS* Chinese DIC scoring system, *DIC* disseminated intravascular coagulation, *WBC*,white blood cell, *BM* bone marrow, *FAB* French, American, British, *AT* antithrombin, *Alb* albumin, *Crea* creatinine, *HDL* high-density lipoprotein, *Glu* glucose, *CK* creatine kinase, *CK-MB* creatine kinase isoenzyme MB, *SF* serum ferritin, *Myo* myoglobin, *HR* hazard ratio, *CI* confidence interval

### Subgroup analyses

**Fig. 3:**
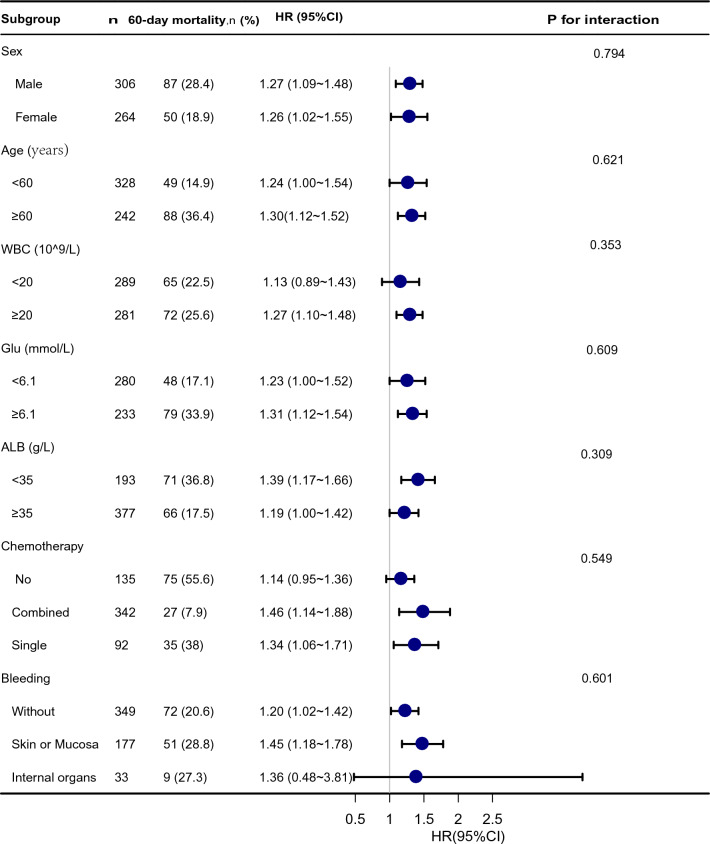
Stratified analyses of the association between CDSS score and 60-day mortality according to baseline characteristics**. **Note: The *p-*value for interaction represents the likelihood of interaction between the variable and the CDSS score. WBC was entered as a categorical variable. The event is so less in thrombosis subgroup, that it was not shown. Adjusts for: sex + age + WBC + AT + Alb + Myo + Glu + chemotherapy + bleeding or thrombosis; *CDSS* Chinese DIC scoring system, *DIC* disseminated intravascular coagulation, *WBC* white blood cell, *Glu* glucose, *Alb* albumin, *HR* hazard ratio, *CI* confidence interval

Analyses and interactive analyses were stratified according to confounders to detect whether the association between CDSS score and 60-day mortality of AML was present in different subgroups. The confounders that were confirmed after a univariate analysis of risk factors (Table S2) and previous findings and clinical constraints, included age, sex, WBC, Glu, ALB, chemotherapy and bleeding (Fig. 3). No significant interaction was observed in any subgroups (P-value for interaction > 0.05).

### Cause of 60-day mortality

There were 137 patients suffered from 60-day mortality, the main causes were organ failure, hemorrhage or Thrombosis, infection, particular pulmonary infection (Table 3).

**Table 3:** Analysis of cause of 60-day mortality in 137 patients with non-APL AML

Cause of death	*N*	%
Organ failure		
Multiple organ failure	7	5.11
Respiratory failure^a^	11	8.03
Cardiac failure	10	7.30
Hemorrhage or Thrombosis		
DIC^b^	8	5.84
Cerebral hemorrhage	11	8.03
Cerebral infarction	6	4.38
Other^c^	3	2.19
Infection		
Sepsis	4	2.92
Pulmonary infection^d^	47	34.31
Other organ^e^	8	5.84
Others	22	16.06

*DIC* disseminated intravascular coagulation

^a^ Nine of the 11 patients also suffered from pulmonary infection

^b^ Of the eight patients, 3 suffered from cerebral hemorrhage, 1 renal hemorrhage and 1 scrotal1hemorrhage

^c^These three patients were suffered form myocardial infarction, spleen infarction,and gastrointestinal hemorrhage, respectively

^d^Three of the 47 patients also suffered from other site infection, including 2 cases gum and 1 soft tissue

^e^Other sites of infection included the mouth, tonsils and tissues, etc

## Discussion

This paper reports a cohort study on the association between CDSS score and 60-day mortality in patients with primary AML. In this real-world study, we evaluated Chinese patients with primary AML using the CDSS criteria and found that 10.4% of patients met the overt DIC criteria (CDSS ≥ 6), similar to a previous report [16]. Among patients with overt DIC, the 60-day all-cause death rate was as high as 47.5%, over twice that of patients with non-overt DIC (21.3%). The risk increased with an increase in the CDSS score, independent of sex, age, and other confounders. The results remained robust with no or gradual adjustments. These findings illustrate that physicians should pay more attention to DIC screening in patients with primary AML and start timely intervention and early treatment to improve patient outcomes. We also noted that patients with elevated leukocyte counts (WBC ≥ 20 × 10^9^/L) were more likely to develop DIC, as was AML-M5, as previously reported [24]. Abnormal frequencies of DD and PLT are higher in CDSS laboratory integral terms, so special attention should be paid to these two items in the diagnosis of AML secondary DIC. It has long been known that PT and APTT are normal in more than half of AML patients with DIC [12].APTT was added to the CDSS integration system to improve the sensitivity of DIC. In our study, 40.7% (24 cases) of overt DIC patients had prolongation of PT (≥ 3S) or 4.1% (4 cases) prolongation of APTT ≥ 10 s, respectively. The results are consistent with those of previous reports; however, the abnormal APTT rate is much lower among those patients. In a prospective study, Wu et al. explored the association between CDSS scores and 28-day all-cause mortality in 753 patients (including sepsis/severe infection, trauma or surgery, and solid tumors, among others). Their results demonstrated that the CDSS DIC score was an independent predictor of mortality [25]. However, they neither included patients with AML nor adjusted for confounders. Additionally, we observed that the mean age of patients with AML was 51.8 years, much younger than that of the European population (65 years) [4].

In CDSS criteria, platelet is different between patients with Hematologic neoplasms and Non-Hematologic neoplasms,compared to ISTH criteria, and D-dimer included clear concentration criteria, while ISTH criteria included judgement of no, slightly elevated and significantly elevated. So, These refinement may be easier for clinicians. In multivariate Cox regression, the CDSS and ISTH scores, as continuous or binary variables, HRs and 95% CIs were similar; however, the former HRs were slightly higher and the laboratory items are more widespread in China.

Hematologists usually evaluate the risk of early mortality based on the clinical performance status and laboratory data. However, the definition of early mortality in AML was different; it was defined as death within 60 days from diagnosis or at the start of chemotherapy [7, 8]. A previous study reported an early mortality rate of 21.0–37.5% [5, 7, 8, 26]. Our results, which showed mortality within 60 days after admission was 24.0%, are similar to those of previous studies. Furthermore, 60-day mortality or early mortality remains an essential clinical problem, which is the critical period for successful clinical management of patients with AML; however, the causes are complicated and unknown, even though hematologists have been trying to reduce its risk. Our study differs slightly from previous studies. Most studies only included patients who had received chemotherapy [27, 28]; the others were excluded due to poor conditions [8]. DIC is a common and severe complication of critical conditions and is highly life-threatening [15], it also is one of the most common causes of severe intracerebral or pulmonary hemorrhage, and AML may trigger DIC. A shift in the thinking process to recognize DIC early in its progression is stressed to help treat better these patients in the future [12].

This study had several noteworthy limitations. First, DIC is a dynamic pathological process, so "dynamic monitoring" is essential for DIC diagnosis, especially regarding chemotherapy duration. We collected admission data within 48 h, so the findings of this research only reflect the period before anti-leukemia treatment. Second, although these findings raise questions about potential 60-day mortality, interpretation is limited by the observational study design; therefore, we could not have a direct risk of CDSS score for patients with AML. Third, the data on fibrin degradation products (FDP) were not available for most cases, so we could not adjust FDP in multivariate Cox regression analysis; however, we compared the CDSS score to the ISTH score and found that the hazard ratios (HRs) were similar between the two scoring systems in the crude model or different adjusted models. Furthermore, this is a retrospective study; the data were collected from 2013 to 2021, and the date of death data for some patients was obtained by telephone follow-up and may be biased. To reduce bias, we conducted interviews with at least two or three family members to determine the exact survival time of the patients. Moreover, different batches of experimental reagents may affect the test results of the CDSS project to some degree. To make the values dependable, IQC was required every day to ensure that the results were under control before testing the clinical specimens. We also participated in an external quality assessment (EQA) organized by the NCCL two times per year to ensure the accuracy of the testing results. Fortunately, they satisfied both the control results. Meanwhile, the instrument must undergo calibration twice yearly as part of regular maintenance. Therefore, all the testing results were dependable.

## Conclusion

The CDSS score may be closely associated with 60-day mortality in patients with primary AML. These results, which are of great clinical significance in patients with CDSS scores ≥ 6, suggest that hematologists should quickly start anti-DIC and anti-leukemia treatments. However, further research is required to confirm and validate these associations.

## Supplementary Information

Below is the link to the electronic supplementary material.

Supplementary file1 (DOCX 15 KB)

Supplementary file2 (DOCX 16 KB)

Supplementary file3 (DOCX 15 KB)

## Data Availability

The raw data required to reproduce these findings cannot be shared at this time, as the data forms part of an ongoing study. However, if necessary, some or all the data generated or used during the study are available from the corresponding author upon request.
