# Supplementary material for: Association of CDSS score and 60-day mortality in Chinese patients with non-APL acute myeloid leukemia: a retrospective cohort study
Source: J Thromb Thrombolysis. 2023 Jun 23;56(3):423–32. doi: 10.1007/s11239-023-02850-6 (PMC10439046; doi:10.1007/s11239-023-02850-6)
Supplement: Supplementary file 1 — Supplementary file1 (DOCX 15 KB) [file 11239_2023_2850_MOESM1_ESM.docx]

| **Table S1** Criteria for CDSS scoring systems | | |
| --- | --- | --- |
| **Establish** | **If** | **Point(s)** |
| Underlying disease | Present | 2 |
| Clinical presentation， meet any one |  |  |
|  | abnormal bleeding | 1 |
|  | unexplained organ failure | 1 |
|  | shock or microcirculatory disorder independent of original disease | 1 |
| Laboratory test |  |  |
| PLT, in patients with non-hematologic neoplasms |  |  |
|  | ≥100×109/L | 0 |
|  | ≥80，but<100×109/L | 1 |
|  | <80×109/L | 2 |
|  | ≥50% decrease within 24 hrs | 1 |
| PLT，in patients with hematologic neoplasms |  |  |
|  | <50×109/L | 1 |
|  | ≥50% decrease within 24 hrs | 1 |
| D-dimer |  |  |
|  | <5 mg/L | 0 |
|  | ≥5,but <9 mg/L | 2 |
|  | ≥9 mg/L | 3 |
| Prolongation of PT and APTT |  |  |
|  | PT＜3s and APTT＜10s | 0 |
|  | PT≥3s or APTT≥10s | 1 |
|  | PT≥6s | 2 |
| Fg |  |  |
|  | ≥1 g/L | 0 |
|  | <1 g/L | 1 |
| **Notes**: in patients with non-hematologic neoplasms,score everyday, if CDSS score ≥7points , overt-DIC present; in patients with hematologic neoplasms,clinical presentation do not count,score everyday, if CDSS score ≥6 points, overt-DIC present. | | |
| **Abbreviations**: CDSS, Chinese DIC scoring system; DIC, disseminated intravascular coagulation; PLT，platelet；DD,D-dime;PT, prothrombin time; APTT, activated partial thromboplastin time; Fg, fibrinogen. | | |
