# Supplementary material for: Association of CDSS score and 60-day mortality in Chinese patients with non-APL acute myeloid leukemia: a retrospective cohort study
Source: J Thromb Thrombolysis. 2023 Jun 23;56(3):423–32. doi: 10.1007/s11239-023-02850-6 (PMC10439046; doi:10.1007/s11239-023-02850-6)
Supplement: Supplementary file 2 — Supplementary file2 (DOCX 16 KB) [file 11239_2023_2850_MOESM2_ESM.docx]

| **Table S2** Univariate analysis of risk factor associated with 60-day mortality in patients with AML | | |
| --- | --- | --- |
| **Variables** | **HR (95%CI)** | ***P*-Value** |
| Sex |  |  |
| Male | Ref. |  |
| Female | 0.62 (0.44,0.88) | 0.007 |
| Age | 1.04 (1.03,1.05) | < 0.001 |
| FAB |  |  |
| AML-M2 | Ref. |  |
| AML-M5 | 0.93 (0.63,1.38) | 0.717 |
| Other | 1.23 (0.81,1.87) | 0.321 |
| Chemotherapy |  |  |
| Without | Ref. |  |
| Combined | 0.10 (0.06,0.15) | < 0.001 |
| Single | 0.58 (0.39,0.86) | 0.007 |
| Pulmonary infection, n (%) | |  |
| No | Ref. |  |
| Yes | 1.07 (0.75,1.52) | 0.705 |
| Genomic risk category, n (% | |  |
| Low | Ref. |  |
| Medium | 2.41 (1.12,5.19) | 0.025 |
| High | 1.86 (0.83,4.18) | 0.133 |
| Bleeding, n (%) |  |  |
| Without | Ref. |  |
| Skin or Mucosa | 1.47 (1.02,2.1) | 0.037 |
| Internal organs | 2.44 (0.99,6.04) | 0.054 |
| Thrombosis | 1.46 (0.73,2.91) | 0.288 |
| WBC | 1.003 (1.00,1.01) | < 0.001 |
| PLT | 0.99 (0.99,1.00) | 0.019 |
| APTT | 1.01 (1,1.02) | 0.008 |
| Fg | 0.94 (0.84,1.06) | 0.32 |
| DD | 1.01(1.00,1.01) | 0.033 |
| AT | 0.97 (0.96,0.98) | < 0.001 |
| ALB | 0.90(0.87,0.93) | < 0.001 |
| Crea | 1.0024 (1.00,1.00) | < 0.001 |
| TG | 0.71 (0.53,0.96) | 0.027 |
| HDL | 0.38 (0.19,0.75) | 0.005 |
| Glu | 1.1 (1.04,1.15) | < 0.001 |
| CK-MB | 1.0072 (1.0,1.02) | 0.094 |
| SF | 1.00 (1.00,1.00) | 0.007 |
| Myo | 1.00 (1.00,1.01) | < 0.001 |
| BM blast | 1.00 (0.9,1.01) | 0.698 |
| CDSS | 1.39 (1.25,1.54) | < 0.001 |
| **Abbreviations:** FAB, French, American, British; WBC, white blood cell; PLT，platelet； BM, bone marrow. PT, prothrombin time; APTT, activated partial thromboplastin time; Fg, fibrinogen; DD, D-dime; AT, antithrombin; ALB, albumin; Crea, creatinine; TG, triglyceride; HDL, high-density lipoprotein; Glu, glucose; CK-MB, creatine kinase isoenzyme MB; SF, serum ferritin; Myo, myoglobin; CDSS, Chinese DIC scoring system; DIC, disseminated intravascular coagulation. | | |
