# Supplementary material for: Association of CDSS score and 60-day mortality in Chinese patients with non-APL acute myeloid leukemia: a retrospective cohort study
Source: J Thromb Thrombolysis. 2023 Jun 23;56(3):423–32. doi: 10.1007/s11239-023-02850-6 (PMC10439046; doi:10.1007/s11239-023-02850-6)
Supplement: Supplementary file 3 — Supplementary file3 (DOCX 15 KB) [file 11239_2023_2850_MOESM3_ESM.docx]

**Table S3** Multivariate Cox regression for ISTH score on 60-day mortality of AML

| **Variables** | **Non-adjusted Model** | |  | **Model Ⅰ** | |  | **Model Ⅱ** | |  | **Model Ⅲ** | |
| --- | --- | --- | --- | --- | --- | --- | --- | --- | --- | --- | --- |
|  | HR (95% CI) | *P*-value |  | HR (95% CI) | *P*-value |  | HR (95% CI) | *P-*value |  | HR (95% CI) | *P-*value |
| ISTH score | 1.33 (1.22~1.46) | <0.001 |  | 1.31 (1.20~1.13) | <0.001 |  | 1.20 (1.03~1.39) | 0.016 |  | 1.26 (1.01~1.54) | 0.037 |
| Binary variable | |  |  |  |  |  |  |  |  |  |  |
| ISTH<5 | Ref. |  |  | Ref. |  |  | Ref. |  |  | Ref. |  |
| ISTH≥5 | 2.47 (1.70~3.58) | <0.001 |  | 2.19 (1.50~3.19) | <0.001 |  | 2.27 (1.27~4.05) | 0.001 |  | 2.74(1.26~5.95) | 0.017 |

**Notes:** Model Ⅰ: Adjusts for sex+ age; Model Ⅱ:adjusts for Model Ⅰ+ FAB subtype+ bleeding+ prognostic stratification+ Pulmonary infection+ chemotherapy；Model Ⅲ: adjusts for Model Ⅱ+ BM blast+ AT+ ALB+ Crea+ HDL+ TG+ Glu+ CK-MB+ Myo+ SF+WBC. Dummy variables were used for SF; WBC was used as a binary variable.

**Abbreviations:** CDSS, Chinese DIC scoring system; DIC, disseminated intravascular coagulation; WBC, white blood cell; BM, bone marrow. FAB, French, American, British; AT, antithrombin; Alb, albumin; Crea, creatinine; HDL, high-density lipoprotein; Glu, glucose; CK, creatine kinase; CK-MB, creatine kinase isoenzyme MB; SF, serum ferritin; Myo, myoglobin. HR, hazard ratio; CI, confidence interval.
